# High Precision Position Measurement Method for Laguerre-Gaussian Beams Using a Quadrant Detector

**DOI:** 10.3390/s18114007

**Published:** 2018-11-16

**Authors:** Qian Li, Jiabin Wu, Yunshan Chen, Jingyuan Wang, Shijie Gao, Zhiyong Wu

**Affiliations:** 1 Changchun Institute of Optics, Fine Mechanics and Physics, Chinese Academy of Sciences, Changchun 130033, China; wujiabin777@163.com (J.W.); yiyunsn@163.com (Y.C.); wangjy_ciomp@163.com (J.W.); yoursjohn@163.com (S.G.); wuzy@ciomp.ac.cn (Z.W.); 2 University of Chinese Academy of Science, Beijing 100049, China

**Keywords:** position measurement method, quadrant detector, Laguerre-Gaussian beams, least square method, effective radius, detection

## Abstract

In this paper, we propose a new method to improve the position measurement accuracy for Laguerre-Gaussian beams on a quadrant detector (QD). First, the error effects of the detector diameter and the gap size are taken into account, and the position error compensation factor is introduced into the conventional formula. Then, in order to reduce the number of parameters, the concept of effective radius is proposed. Thus, a new analytical expression is obtained with a best fit using the least square method. It is verified by simulation that this approach can reduce the maximum error by 97.4% when the beam radius is 0.95 mm; meanwhile, the root mean square errors under different radii are all less than 0.004 mm. The results of simulation show that the new method could effectively improve the accuracy of the QD measurement for different radii. Therefore, the new method would have a good prospect in the engineering practice of beam position measurements.

## 1. Introduction

The Laguerre-Gaussian (L-G) beams possessing orbital angular momentum (OAM) are becoming a hot topic in academia [1,2,3,4]. Recent theoretical developments have revealed that using the L-G beams can greatly improve the information capacity of wireless optical communication systems [5]. The position measurement technology of the L-G beams plays an important role in an OAM wireless optical communication system using multiplexing of OAM beams since the misalignment of the beam may lead to power leakage, thus reducing the probability of the receiver to detect the OAM state correctly. Furthermore, the position measurement technology of the L-G beams has also been widely applied in many areas such as optical tweezing [6,7,8,9] and micromanipulation [10].

Compared with position sensitive detector (PSD) and charge-coupled devices (CCD), the quadrant detector (QD) is suitable for position measurement of the L-G beams, owing to its high resolution and fast response [11]. However, the problem with such an implementation is that there is a non-linear relationship between the detector output signal and the centroid position of the beams [12]. It is mainly due to the intensity distribution of the beams and the inhomogeneity of the detector shape. As a result, there is a lower accuracy when the beam center is far from the QD origin since the beam center is outside the linear working interval [13]. N. Hermosa et al. proved that when the angular quantum index is high, the QD response to the L-G beams can be approximated by its response to hard-ringed beams [14]. Valeria Garbin et al. showed the potential for position detection of dielectric particles using L-G beams with a QD configuration [15]. With the purpose of enhancing linearity, Song Cui et al. constructed a new solution equation achieving better measurement accuracy [16,17]. However, these approaches introduce a lot of parameters and, therefore, they are time-consuming. Infinite integral method (IIM) is a solution which has a good ability to suppress nonlinearity. Nevertheless, IIM is suffering from its low accuracy [18,19]. Such a low accuracy cannot be ignored especially to an OAM wireless optical communication system using multiplexing of OAM beams, since the low inherent crosstalk and power-coupling loss generally rely on accurate on-axis measurement of the multiple OAM beams [20,21]. In brief, there is an urgent need for a method with high precision and less parameters.

In this study, we deeply analyze the relationship between the detector output signal and the centroid position of the L-G beams in different modes. The error compensation factor is introduced to compensate for the influence of detector diameter and gap size based on the IIM. Then the effective beam radius is obtained by the least square fitting method, and thus a new position measurement method is proposed. The accuracy of the new method is evaluated by simulation. The results show that the proposed method can effectively improve the position measurement accuracy of the L-G beams in a wide measurement range.

The rest of the paper is organized as follows: We start our paper by presenting the intensity distribution of the L-G beams and position measurement principle of the QD in Section 2.1. In Section 2.2, IIM is described, whereafter, the limitations of IIM dealing with L-G beams are briefly discussed. Then, the improved new estimation method is proposed. We also demonstrate the feasibility of the improved new estimation method under different radii by simulation in Section 3. Finally, conclusions are drawn in Section 4.

## 2. Materials and Methods

### 2.1. Theoretical Analyses of L-G Beams and Quadrant Detector

#### 2.1.1. Intensity Distribution of L-G Beams

Unlike a general beam whose wavefront is a plane or sphere, the power intensity distribution for L-G beams is:

(1) $$D_{p}^{l}(r)=\frac{K^{2}p!}{(p+|l|)!\omega^{2}}\exp\left(\frac{-2r^{2}}{\omega^{2}}\right){\left(\frac{2r^{2}}{\omega^{2}}\right)}^{\left|l\right|}{\left[L_{p}{}^{\left|l\right|}\left(\frac{2r^{2}}{\omega^{2}}\right)\right]}^{2}$$

where r is the distance from the beam center, ω is the beam radius, $L_{p}^{\left|l\right|}(x)$ is the Laguerre generalized polynomial, p is the radial index, and l is the azimuthal index.

**Figure 1:**
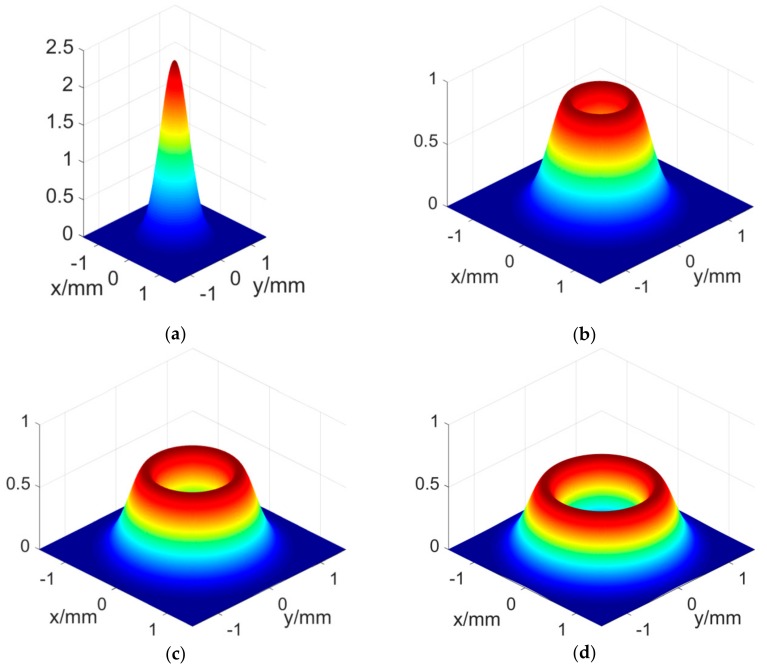
Power intensity distribution of the L-G beams (K=1, ω=0.65mm ), (**a**) p = 0, l = 0, (**b**) p = 0, l = 1, (**c**) p = 0, l = 2, (**d**) p = 0, l = 3.

As shown in Figure 1a, when p=l=0, the L-G beams degenerate into a Gauss beam whose power concentrates on the center:

(2) $$D_{0}^{0}(x,y)=\frac{K^{2}\exp\left(-2\frac{{\left(x-x_{0}\right)}^{2}+(y-y_{0}{}^{2})}{\omega^{2}}\right)}{\omega^{2}}.$$

When p≠0 or l≠0, the L-G beams have annular energy distributions. Only the case of p=0 is considered in this paper. As shown in Figure 1b–d, with the increase of l, the annular spot gradually expands outward. The power intensity distributions of the L-G beams change as follows:

(3) $$D_{0}^{1}(x,y)=\frac{2K^{2}\exp\left(-2\frac{{\left(x-x_{0}\right)}^{2}+(y-y_{0}{}^{2})}{\omega^{2}}\right)\left({\left(x-x_{0}\right)}^{2}+(y-y_{0}{}^{2})\right)}{\omega^{4}},$$

(4) $$D_{0}^{2}(x,y)=\frac{2K^{2}\exp\left(-2\frac{{\left(x-x_{0}\right)}^{2}+(y-y_{0}{}^{2})}{\omega^{2}}\right){\left({\left(x-x_{0}\right)}^{2}+(y-y_{0}{}^{2})\right)}^{2}}{\omega^{6}},$$

(5) $$D_{0}^{3}(x,y)=\frac{4K^{2}\exp\left(-2\frac{{\left(x-x_{0}\right)}^{2}+(y-y_{0}{}^{2})}{\omega^{2}}\right){\left({\left(x-x_{0}\right)}^{2}+(y-y_{0}{}^{2})\right)}^{3}}{3\omega^{8}}.$$

As can be seen from Figure 1, there is an obvious difference between the Gauss beam and the L-G beams. Unlike a Gauss beam whose power concentrates on the center, the L-G beams have an annular energy distribution which is much more complicated for position measurement.

#### 2.1.2. Position Measurement Principle of the QD

**Figure 2:**
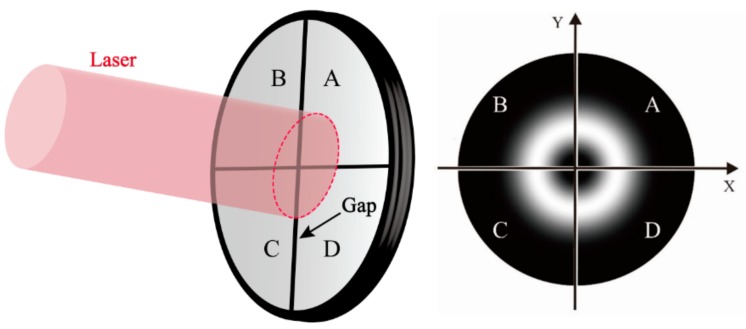
Beams incident on a QD.

The QD can be seen as a device consisting of four identical photodiodes separated by small gaps without photoelectric effect [22], as shown in Figure 2. If there is an incident beam, each quadrant will induce the corresponding photocurrent $I_{i}(i=A,B,C,D)$. We assume that the power intensity distribution of the incident beam is D(x,y), and that the centroid position of the beam is $\left(x_{0},y_{0}\right)$, then:

(6) $$I_{i}=\iint_{S_{i}}D_{p}^{l}(x-x_{0},y-y_{0})dxdy\;\;(i=A,B,C,D).$$

The conventional formulas to estimate the beam position are:

(7) $$E_{X}=\frac{I_{A}+I_{D}-I_{B}-I_{C}}{I_{A}+I_{B}+I_{C}+I_{D}},$$

(8) $$E_{Y}=\frac{I_{A}+I_{B}-I_{C}-I_{D}}{I_{A}+I_{B}+I_{C}+I_{D}}.$$

$E_{X}$ and $E_{Y}$ represent the extent of deviation from the origin of the QD in the x and y directions, respectively. However, $E_{X}$ and $E_{Y}$ are not equal to the centroid position of the beam. In order to accurately locate the position of the beam in real time, we set the upper limit of the integral to the boundary of the QD. Since the shape of the QD and the beam profiles are symmetric, it is expected to achieve the same position measurement results in both the x and y directions. Consequently, only the position measurement results in the x direction are discussed here. The relationship between the estimation and the centroid position can be obtained:

(9) $$E_{X}=\frac{\left(\int_{-A}^{A}\int_{B}^{C}D(x,y)dxdy-\int_{-B}^{B}\int_{B}^{C}D(x,y)dxdy\right)-\left(\int_{-A}^{A}\int_{-C}^{-B}D(x,y)dxdy-\int_{-B}^{B}\int_{-C}^{B}D(x,y)dxdy\right)}{\left(\int_{-A}^{A}\int_{B}^{C}D(x,y)dxdy-\int_{-B}^{B}\int_{B}^{C}D(x,y)dxdy\right)+\left(\int_{-A}^{A}\int_{-C}^{-B}D(x,y)dxdy-\int_{-B}^{B}\int_{-C}^{B}D(x,y)dxdy\right)},$$

where $A=\sqrt{R^{2}-x^{2}}$, B=d/2, $C=\sqrt{R^{2}-d^{2}/4}$, R is the radius of the QD and d is the gap width. Centroid position can be obtained from Equation (9):

(10) $$x_{0}=f^{-1}(\omega ,E_{X},R,d).$$

However, Equation (10) is a transcendental equation that cannot be solved analytically, which will bring great difficulty to practical applications.

### 2.2. Improved New Estimation Method

#### 2.2.1. Infinite Integral Method

A solution is developed in Reference [23], namely the infinite integral method, in which the detector radius is assumed to be large enough, the influence of the gap size is neglected, and the upper limit of the integral is set to infinite. The IIM is used to calculate the estimation as shown in Equation (11):

(11) $$E_{X}(p,l)\approx \frac{\int_{-\infty }^{\infty }\int_{0}^{\infty }D_{p}^{l}(x,y)dxdy-\int_{-\infty }^{\infty }\int_{-\infty }^{0}D_{p}^{l}(x,y)dxdy}{\int_{-\infty }^{\infty }\int_{-\infty }^{\infty }D_{p}^{l}(x,y)dxdy}.$$

Equations (2)–(5) are taken into Equation (11) respectively. Let $t=\frac{\sqrt{2}x_{0}{}^{\prime }}{\omega }$, where $x_{0}{}^{\prime }$ is the approximation of $x_{0}$, thus:

(12) $$E_{X}(0,0)\approx erf(t),$$

(13) $$E_{X}(0,1)\approx erf(t)-\frac{te^{-t^{2}}}{\sqrt{\pi }},$$

(14) $$E_{X}(0,2)\approx erf(t)-\frac{e^{-t^{2}}(2t^{3}+5t)}{4\sqrt{\pi }},$$

(15) $$E_{X}(0,3)\approx erf(t)-\frac{e^{-t^{2}}(4t^{5}+16t^{3}+33t)}{24\sqrt{\pi }}.$$

**Figure 3:**
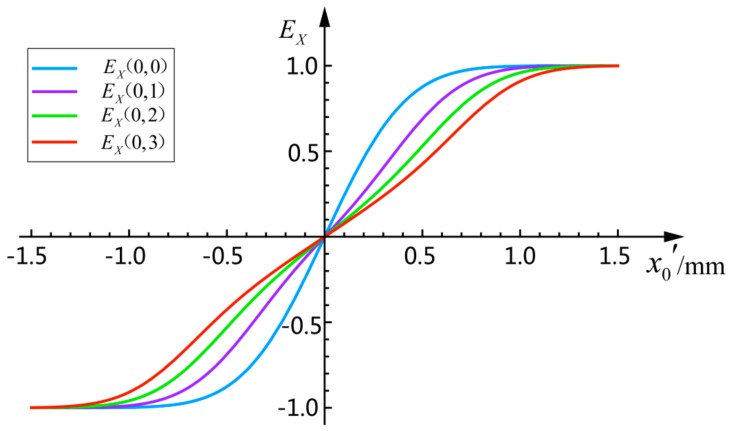
Relationship of $x_{0}{}^{\prime }$, $E_{X}(0,0)$, $E_{X}(0,1)$, $E_{X}(0,2)$, and $E_{X}(0,3)$.

When ω is 0.65 mm, the non-linear relationship of $x_{0}{}^{\prime }$, $E_{X}(0,0)$, $E_{X}(0,1)$, $E_{X}(0,2)$, and $E_{X}(0,3)$ is shown in Figure 3:

Let P(t)=erf(t), then:

(16) $$E_{X}(0,0)\approx P(t)=P(\frac{\sqrt{2}x_{0}{}^{\prime }}{\omega }).$$

We can get the estimation:

(17) $$x_{0}{}^{\prime }=\frac{P^{-1}(E_{X})\omega }{\sqrt{2}}.$$

Which ignores the influence of the detector’s diameter and the gap size. Equation (17) is the expression of IIM. In this paper, we restrict ourselves to $LG_{p=0}^{l=1}$ beam, and the situations of L-G beams with other modes are similar to this.

Similar to the Gauss beam, we assume $Q(t)=erf(t)-\frac{te^{-t^{2}}}{\sqrt{\pi }}$, then:

(18) $$E_{X}\approx Q(t)=Q(\frac{\sqrt{2}x_{0}{}^{\prime }}{\omega }).$$

Thus, the estimation of $LG_{p=0}^{l=1}$ beam can be obtained:

(19) $$x_{0}{}^{\prime }=\frac{Q^{-1}(E_{X})\omega }{\sqrt{2}}.$$

**Figure 4:**
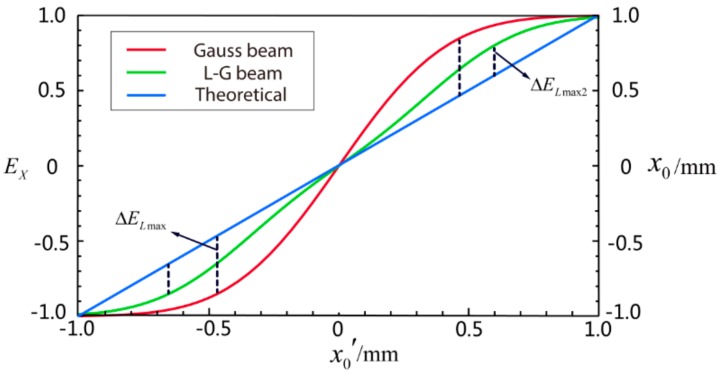
The simulation curves of estimation in the measurement range from −1 mm to 1 mm; the blue line represents the theoretical estimation; the red curve is the estimation of Gauss beam by simulation; and the green curve is the estimation of L-G beam by simulation.

As shown in Figure 4, there is a maximum error in a certain measurement range. The linearity of position measurement using QD in x-direction is defined as:

(20) $$\sigma_{x}=\frac{\left|\Delta E_{L\max}\right|}{S},$$

where $\left|\Delta E_{L\max}\right|$ denotes the maximum error and S is the measurement range. It is obvious that the maximum error of Gauss beam $\Delta E_{L\max}$ is larger than the maximum error of $LG_{p=0}^{l=1}$ beam $\Delta E_{L\max2}$. Therefore, the linearity of the estimation of the $LG_{p=0}^{l=1}$ beam is better than that of the Gaussian beam, which means a higher position measurement accuracy in theory.

**Figure 5:**
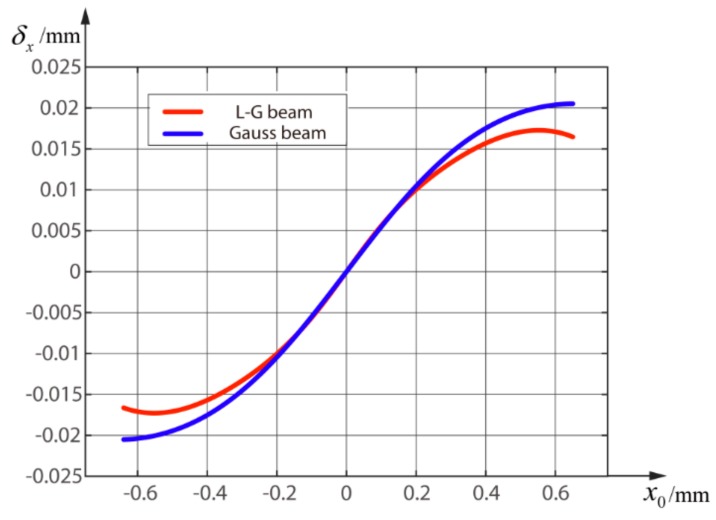
Errors of the $LG_{p=0}^{l=1}$ beam and the Gauss beam using the IIM when ω is 0.65 mm.

In Figure 5, the error $\delta_{x}=x_{0}{}^{\prime }-x_{0}$ increases with the increment of the distance from the origin. As mentioned before, the estimation of the $LG_{p=0}^{l=1}$ beam has a better accuracy than that of the Gauss beam. Even so, the maximum error still reaches 0.016 mm, which will have a great impact on the accuracy of the system. Therefore, it is necessary to improve the IIM.

#### 2.2.2. Improved New Estimation Method

It can be seen that the $x_{0}{}^{\prime }=\frac{Q^{-1}(E_{X})}{\sqrt{2}}\cdot \omega$ in Equation (19) is the product of two parts. $Q^{-1}(E_{X})$ is a function of the $E_{X}$, which determines the overall change trend of the entire function. ω is the radius of the incident beam, which can be seen as the proportional coefficient of the centroid position and the estimation.

In order to obtain a higher accuracy, an error compensation factor η=f(ω,R,d) is introduced in consideration of the error effects of the detector diameter and the gap size. The estimation of the beam position can be written as:

(21) $$x_{0}{}^{\prime }=\frac{Q^{-1}(E_{X})}{\sqrt{2}}\cdot \omega \cdot \eta (\omega ,R,d),$$

where η(ω,R,d) introduces factors R and d which are not considered by IIM. R and d are fixed in a practical application, η(ω,R,d) has only one variable parameter ω. So we combine the last two parameters into one, redefined as the effective beam radius $\omega_{e}=\omega \cdot \eta (\omega ,R,d)$, then:

(22) $$x_{0}{}^{\prime }=G(E_{X})\cdot \omega_{e},$$

where $G(E_{X})=\frac{Q^{-1}(E_{X})}{\sqrt{2}}$. Equation (22) is the expression of the improved new estimation method. The effective beam radius $\omega_{e}$ represents the influence of ω,R,d on the beam position. In order to obtain the $\omega_{e}$, we assume a beam with a radius of ω is incident on a QD with radius *R* and gap width *d*. The beam is moved from (−ω,0) to (ω,0) at intervals of 0.001 mm. *N* sets of data points are measured along the x-direction. The centroid position of the beam $x_{0i}(i=1\ldots N)$ and quadrant output current value $I_{Ai}$, $I_{Bi}$, $I_{Ci}$, $I_{Di}$ are recorded. Then, we set the upper limit of the integral to the detector boundaries and calculate the estimation $E_{Xi}$ of each point according to Equation (9). Thus, we can get the $E_{Xi}$ corresponding to the $x_{0i}$. At the same time, $G(E_{Xi})$ of each point are also obtained. With the least square method, the following mathematical model of residuals is constructed by substituting each pair of $\left[x_{0i}{}^{\prime },\;G(E_{Xi})\right]$ into Equation (22):

(23) $$I(\omega_{e})={\left\|\delta_{x}\right\|}^{2}=\sum_{i=1}^{N}[x_{0i}{}^{\prime }(\omega_{e})-x_{0i}{]}^{2},$$

(24) $$\frac{\partial I}{\partial \omega_{e}}=2\sum_{i=1}^{N}G^{2}(E_{Xi})\cdot \omega_{e}-2\sum_{i=1}^{N}G(E_{Xi})\cdot x_{0i}.$$

Let $\frac{\partial I}{\partial \omega_{e}}=0$, the optimal $\omega_{e}$ can be obtained:

(25) $$\omega_{e}=\frac{2\sum_{i=1}^{N}G(E_{Xi})\cdot x_{0i}}{\sum_{i=1}^{N}G^{2}(E_{Xi})}.$$

**Figure 6:**
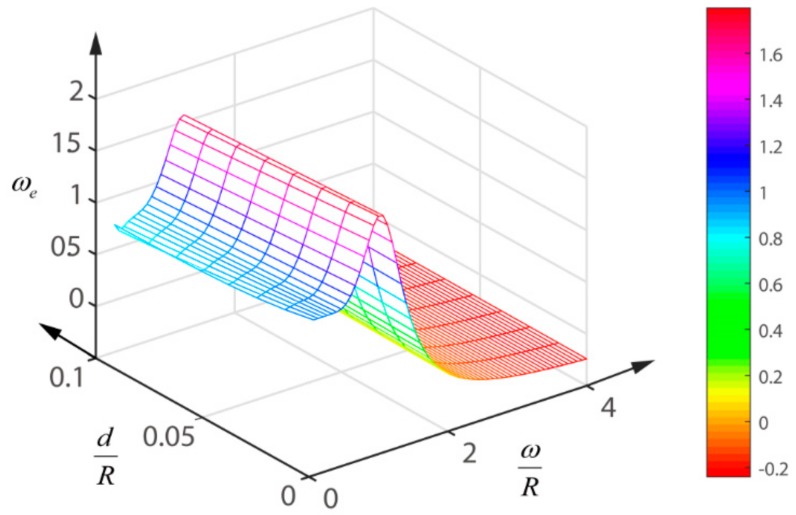
The tendency of $\omega_{e}$ with the changes of $\frac{d}{R}$ and $\frac{\omega }{R}$.

**Figure 7:**
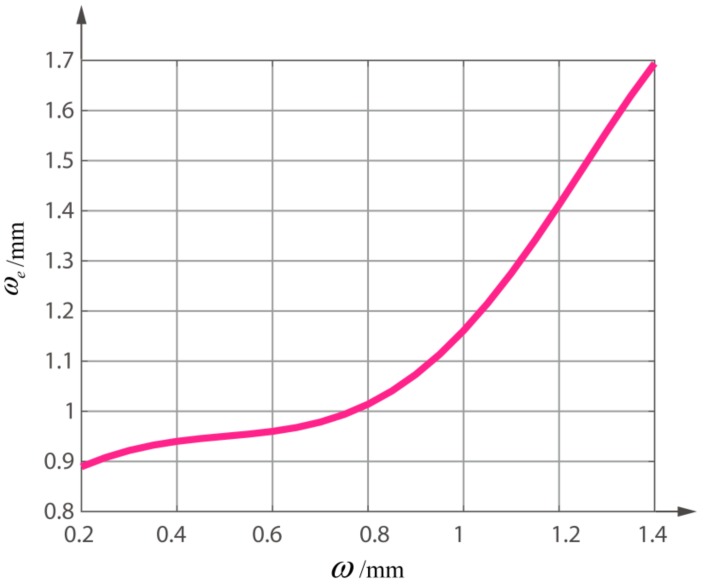
Relationship between ω and $\omega_{e}$ when *R* is 1.5 mm and *d* is 0.045 mm.

In this paper, we use Matlab to simulate the tendency of $\omega_{e}$ with the changes of $\frac{d}{R}$ and $\frac{\omega }{R}$, as shown in Figure 6. As can be seen, $\frac{d}{R}$ has a small effect on the $\omega_{e}$. With the gradual increase of $\frac{\omega }{R}$, a peak of $\omega_{e}$ appears near $\frac{\omega }{R}=1$. In this paper, a QD with *R* = 1.5 mm and *d* = 0.045 mm is discussed as an example. Figure 7 shows the relationship between ω and $\omega_{e}$. As *R* and *d* are fixed, $\omega_{e}$ is only a function of the incident radius ω. Because of the non-linear relationship between $\omega_{e}$ and ω, the polynomial fitting method can be used to fit the expression of $\omega_{e}$. Since the difference of residuals between the six polynomials and the five polynomials is only 0.1 mm, the five polynomial fitting is adopted here, and the expression is as follows:

(26) $$\omega_{e}=-1.0315\omega^{5}+2.9185\omega^{4}-1.7991\omega^{3}-0.402\omega^{2}+0.6992\omega +0.7757.$$

Substituting Equation (26) into Equation (22) results in the polynomial expression of the improved new estimation method:

## 3. Results and Discussion

In order to evaluate the improved new estimation method, the maximum error $\delta_{xMAX}$ and the root mean square error $\delta_{xRMSE}$ are adopted. The specific analysis is as follows.

$\delta_{xMAX}$ is the maximum value of $\left|\delta_{xi}\right|$, which represents the extreme value of the error in the detection range:

(27) $$\delta_{xMAX}=MAX\left(\left|\delta_{xi}\right|\right)=MAX\left(\left|x_{0i}{}^{\prime }(\omega_{e})-x_{0i}\right|\right),$$

$\delta_{xRMSE}$ is the root mean square error (RMSE), which is used to measure the deviation between the observed value and the true value in the detection range:

(28) $$\delta_{xRMSE}=\sqrt{\frac{1}{N}\sum_{i=1}^{N}\delta_{xi}{}^{2}}=\sqrt{\frac{1}{N}\sum_{i=1}^{N}[x_{0i}{}^{\prime }(\omega_{e})-x_{0i}{]}^{2}},$$

**Figure 8:**
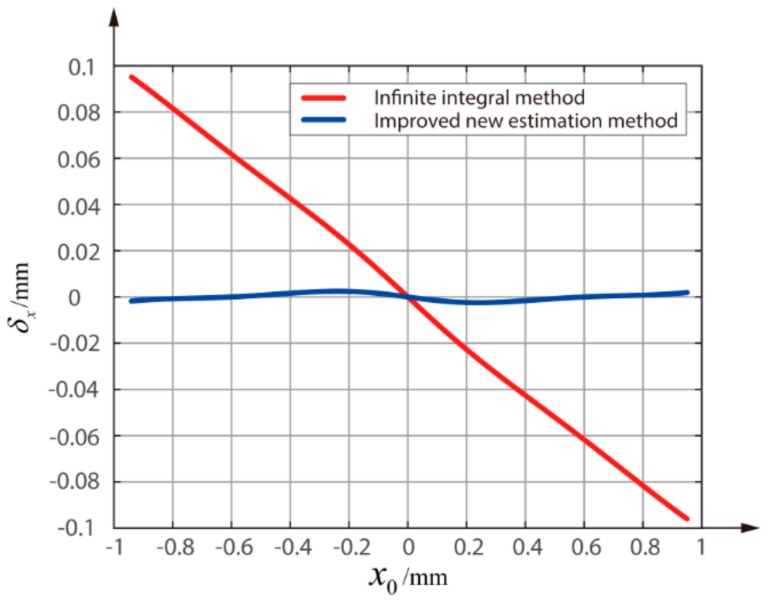
Comparison of the errors between the improved new estimation method and the infinite integral method when ω is 0.95 mm.

When ω is 0.95 mm, $\omega_{e}$ is 1.1124 mm. As shown in Figure 8, the $\delta_{xMAX}$ of the IIM in the detection range of [−0.95~0.95 mm] is 0.096 mm, while the $\delta_{xMAX}$ of the improved new estimation method is 0.0025 mm which is 97.4% lower than the IIM. In addition, the $\delta_{xRMSE}$ of the IIM is 0.0497 mm, while the $\delta_{xRMSE}$ of the improved new estimation method is only 0.0012 mm, which is reduced by 97.6%.

It is worth noting that there are two main types of sources of error in the measurement system: One is the random error caused by the factors such as dark current of the QD and the asymmetry or distortion of the beam; the other is the inherent error existing in the positioning method. The main aim of our method is to eliminate the inherent error. Therefore, a lower signal-to-noise ratio as well as an L-G beam asymmetry or a distortion will affect the position measurement results. In consideration of that, filters should be deployed in the system, and increasing the signal energy is also a good way to improve the signal-to-noise ratio.

**Figure 9:**
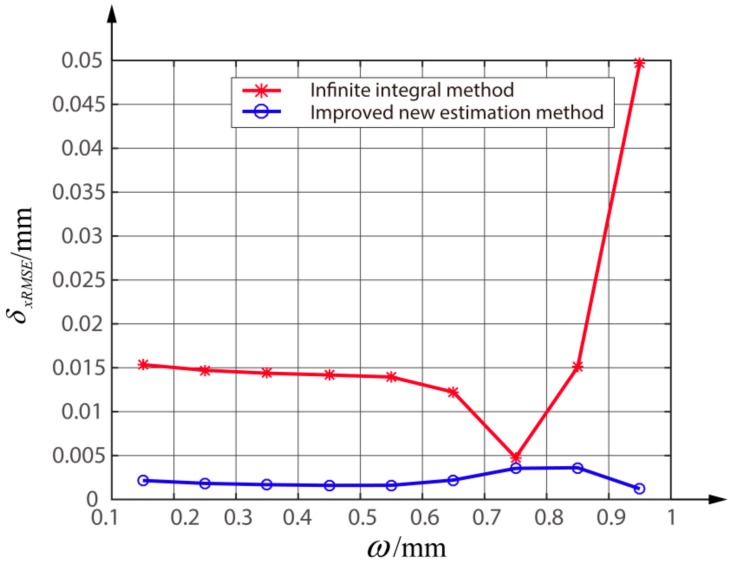
Comparison of the $\delta_{xRMSE}$ between the improved new estimation method and the infinite integral method with different ω.

When we change the ω in the range of [0.15 mm, 0.95 mm], the curves of the $\delta_{xRMSE}$ using IIM and the improved new estimation method are shown in Figure 9. It can be seen that there is a little difference between the $\delta_{xRMSE}$ of the two methods when the radius is near 0.75 mm. This is mainly because an L-G beam with the radius about 0.75 mm is less affected by the gap and avoids too much energy loss. In addition, the $\delta_{xRMSE}$ under different radii are all less than 0.004 mm.

Table 1 compares the $\delta_{xMAX}$ of the two methods with different ω. Similar to the discussion of $\delta_{xRMSE}$, the smallest difference between the $\delta_{xMAX}$ of the two methods is obtained when ω is 0.75 mm. The improved new estimation method has smaller $\delta_{xMAX}$ at different beam radii, all less than 0.01 mm. In brief, the improved new estimation method presented in this paper has better performances both in terms of $\delta_{xMAX}$ and $\delta_{xRMSE}$, and it also shows good stabilities for different beam radii.

For the improved new estimation method, only one parameter effective radius $\omega_{e}$ is introduced. Compared with the method mentioned in Reference [24], the new method has less parameters, which are more suitable for practical applications.

## 4. Conclusions

In conclusion, an improved position measurement method for the L-G beams has been proposed. Through introducing the effective radius, the error effects of the detector diameter and the gap size are compensated. Therefore, compared with IIM, significant accuracy improvement is realized without introducing a large number of parameters. It is verified by simulation that this approach can reduce the maximum error by 97.4% when the beam radius is 0.95 mm; meanwhile, the root mean square errors under different radii are all less than 0.004 mm. The simulation results show the robustness and accuracy of our method with respect to different spot radii. In addition, the proposed method is also applicable for other types of QDs with different radii and gaps. Because of these advantages, this method is expected to be applied in an OAM wireless optical communication system using multiplexing of OAM beams and optical tweezers.

## Figures and Tables

**Table 1:** Comparison of the $\delta_{xMAX}$ between the improved new estimation method and the infinite integral method for different radii.

ω/mm	$\omega_{e}/\mathrm{mm}$	$\delta_{xMAX}\;\mathrm{of}\;\mathrm{the}\;\mathrm{Infinite}\;\mathrm{Integral}\;\mathrm{Method}/\mathrm{mm}$	$\delta_{xMAX}\;\mathrm{of}\;\mathrm{the}\;\mathrm{Improved}\;\mathrm{New}\;\mathrm{Estimation}\;\mathrm{Method}/\mathrm{mm}$	$\mathrm{Decrease}\;\mathrm{Percententage}\;\mathrm{of}\;\mathrm{the}\;\delta_{xMAX}$
0.15	0.9057	0.0225	0.0047	79.1%
0.25	0.9216	0.0227	0.0039	82.8%
0.35	0.9359	0.0228	0.0036	84.2%
0.45	0.9463	0.0228	0.0035	84.6%
0.55	0.9556	0.0223	0.0037	83.4%
0.65	0.9696	0.0173	0.0057	67.1%
0.75	0.9957	0.0095	0.0076	25.0%
0.85	1.0414	0.0419	0.0097	76.8%
0.95	1.1124	0.0960	0.0025	97.4%
